# Blood Interferon-α Levels and Severity, Outcomes, and Inflammatory Profiles in Hospitalized COVID-19 Patients

**DOI:** 10.3389/fimmu.2021.648004

**Published:** 2021-03-09

**Authors:** Marco Contoli, Alberto Papi, Luca Tomassetti, Paola Rizzo, Francesco Vieceli Dalla Sega, Francesca Fortini, Francesca Torsani, Luca Morandi, Luca Ronzoni, Ottavio Zucchetti, Rita Pavasini, Alberto Fogagnolo, Carlo Alberto Volta, Nathan W. Bartlett, Sebastian L. Johnston, Savino Spadaro, Gianluca Campo

**Affiliations:** ^1^Respiratory Section, Department of Translational Medicine, University of Ferrara, Ferrara, Italy; ^2^Azienda Ospedaliera Universitaria Ferrara, Ferrara, Italy; ^3^Department of Physics and Earth Sciences University of Ferrara, Ferrara, Italy; ^4^National Institute of Nuclear Physics, INFN Sezione di Ferrara, Ferrara, Italy; ^5^Laboratory for Technologies of Advanced Therapies, University of Ferrara, Ferrara, Italy; ^6^Maria Cecilia Hospital, GVM Care & Research, Cotignola, Italy; ^7^Cardiology Unit, Azienda Ospedaliero-Universitaria di Ferrara, Cona, Italy; ^8^Intensive Care Unit, Department of Translational Medicine, University of Ferrara, Ferrara, Italy; ^9^Priority Research Centre for Healthy Lungs, University of Newcastle and Hunter Medical Research Institute, Newcastle, NSW, Australia; ^10^National Heart and Lung Institute, Imperial College London, London, United Kingdom

**Keywords:** interferon, SARS–CoV−2, COVID−19, respiratory failure, mortality

## Abstract

**Background:** Deficient interferon responses have been proposed as one of the relevant mechanisms prompting severe manifestations of COVID-19.

**Objective:** To evaluate the interferon (IFN)-α levels in a cohort of COVID-19 patients in relation to severity, evolution of the clinical manifestations and immune/inflammatory profile.

**Methods:** This is prospective study recruiting consecutive hospitalized patients with respiratory failure associated with SARS-COV-2 infection and matched controls. After enrollment, patients were assessed every 7 ± 2 days for additional 2 consecutive visits, for a total of 21 days. The severity of the clinical condition was ranked based on the level of respiratory support required. At each time-point blood samples were obtained to assess immune cells and mediators by multiplex immunoassay.

**Results:** Fifty-four COVD-19 and 11 control patients matched for severity were enrolled. At recruitment, lower levels of blood IFN-α were found in COVID-19 patients compared to controls (3.8-fold difference, *p* < 0.01). Improvements in COVID-19 severity were paralleled by a significant increase of blood IFN-α levels. A significant increase in blood IFN-α was found over the study period in survivors (70% of the study population). A similar trend was found for blood IFN-β with IFN-β levels below the threshold of detectability in a substantial proportion of subjects. Significantly higher values of blood lymphocytes and lower levels of IL-10 were found at each time point in patients who survived compared to patients who died. In patients who clinically improved and survived during the study, we found an inverse association between IL-10 and IFN-α levels.

**Conclusion:** The study identifies a blood immune profile defined by deficient IFN-α levels associated with increased IL-10 expression in patients progressing to severe/life threatening COVID-19 conditions, suggesting the involvement of immunological pathways that could be target of pharmacological intervention.

**Clinical Trial Registration:** ClinicalTrials.gov identifier NCT04343053.

## Introduction

Coronavirus disease (COVID)-19 is a heterogeneous condition caused by Severe Acute Respiratory Syndrome-coronavirus 2 (SARS-COV-2) infection. Clinically, it is generally characterized by an interstitial pneumonia that can lead to impaired gas-exchange, acute respiratory failure and death (1). The pathogenesis is complex and a variable combination of hyper-inflammatory responses and alterations in the coagulation asset have been described in critically hill patients (2, 3). Understanding the immune-inflammatory mechanisms related to the clinical manifestations of COVID-19 can guide the identification of potential pharmacological targets. Deficient immune response, and in particular interferon responses have been proposed as an immunologic mechanism prompting to severe COVID-19 clinical expression (4, 5). We prospectively evaluated interferon (IFN)-α (type I IFN) levels in a cohort of hospitalized COVID-19 patients with respiratory failure and we followed the evolution of the clinical manifestations of the clinical conditions in parallel with immune/inflammatory profile variations. In parallel, blood IFN-α, and immunoinflammatory biomarkers were assessed and compared in a group of control hospitalized subjects with respiratory failure not associated with SARS-COV-2 infection.

## Methods

The study was an investigator-initiated, prospective, single-center study recruiting consecutive patients admitted to Respiratory and Intensive Care Units of the Azienda Ospedaliera Universitaria di Ferrara (Ferrara, Italy) (Clinicaltrials.gov identifier NCT04343053). Patients were included if they had SARS-COV-2 infection (confirmed by PCR-positive nasopharyngeal swab specimens) and respiratory failure [defined as arterial oxygen tension of <8.0 kPa (60 mmHg) at room air and oxygen saturation <90%]. Patients were recruited from April 1 until the end of May 2020. To compare the immunoinflammatory profile of COVID-19 with non-COVID-19 patients a control group of patients, hospitalized in the same period and hospital settings with acute respiratory failure due to respiratory/cardiovascular acute conditions and not related to SARS-COV-2, was also included. An electronic case report form was used to collect anonymized demographic, clinical, laboratory and imaging data. Standard of care treatment, based on best available evidence, was used. After enrollment (T1—Baseline), COVID-19 patients were assessed every 7 ± 2 days for additional 2 consecutive visits (T2 and T3). At each time point, in line with the World Health Organization Ordinal Scale for Clinical Improvement (6), the level and changes of severity were identified on the basis of the type of intervention [3 steps scale: (1) oxygen supplementation; (2) high-flow oxygen or non-invasive mechanical ventilation (NIMV); (3) invasive mechanical ventilation (IMV)]. Furthermore, an increase of 50% or above of the ratio of arterial oxygen partial pressure (PaO_2_ in mmHg) to fractional inspired oxygen (FiO_2_ expressed as a fraction) (PaO_2_/FiO_2_ ratio) was considered a clinically relevant improvement. Study blood samplings were performed from an antecubital vein using a 21-gauge needle. All patients underwent blood sampling early in the morning before treatment administration. Baseline samples were obtained in all patients and COVID-19 patients were also sampled at each subsequent time-points.

Blood samples were analyzed for: (i) inflammatory cell counts (performed at the central laboratory of the Azienda Ospedaliera Universitaria Ferrara, Italy). and (ii) immune mediators including interleukin (IL)-5, IL-6, IL-10, IL-13, IL-1ra, IFN-α, IFN-γ and TNF-α by multiplex immunoassay-based (EMD Millipore Burlington, MA, USA). Data were analyzed by MAGPIX system provided with the xPONENT Software following manufacturer's instructions (Luminex, Thermo Fisher Scientific, Waltham, MA, USA). The levels of blood IFN-β were also measured using commercially available ELISA assay, according to manufacturer's instruction (PBL Assay Science catalog number 41415-1; NJ, USA−1.2 pg/ml LLOQ sensitivity). The immune mediators were assessed in plasma samples. For plasma preparation, the blood samples were collected using 6-ml EDTA-containing tubes. The tubes were centrifuged for 15 min at 2200 RPM. All blood specimens were processed immediately for plasma collection and aliquots were stored at −80°C.

This is a longitudinal, prospective proof-of-concept observational study. At the time of study design (March 2020) no study was available to be used for a formal sample size calculation. The sample size adopted in in the study was sufficient to find significant differences in subsequent studies published on the same topic (7, 8). Patient characteristics were analyzed using Fisher exact, Chi-square or Wilcoxon rank-sum test as appropriate for categorical and continuous variables. Biomarker concentrations were analyzed using Kruskal-Wallis tests followed by Dunn *post hoc* tests when appropriate or pairwise-Wilcoxon tests. A 2-sided *p* < 0.05 was considered statistically significant. Partial correlations among marker concentrations are estimated as the correlation between two variables after adjusting for the (linear) effect of one or more variables and are represented as Gaussian Graphical Models (GGM). Analyses were performed using R 4.0.2 (R Foundation for Statistical Computing, Vienna, Austria. URL https://www.R-project.org/), and the R packages: ggpubr, gtsummary, correlation, ggraph. The protocol was approved by the local ethics committee. Patients consented to participate or their next of kin provided oral consent.

## Results

### Study Population

Fifty-four COVID-19 and 11 control patients were enrolled in the study. The main reasons for hospitalization with respiratory failure in the control group were respiratory (pulmonary infiltrates/pneumonia) and cardiovascular (heart failure). The two groups of patients were matched for age, gender, BMI, smoking history, number of comorbidities per patient and for the need of respiratory support at recruitment (Table 1). None of the patients had prior immunological insufficiencies and none were receiving immunosuppressive medication and/or chemotherapy. The two groups of patients were comparable also in term of outcomes/short term prognosis: 29.6% of the COVID-19 patients and 27.2% of the control patients died during the study period (*p* = 0.87). In line with previous reports, the COVID-19 patients who died were significantly older and with higher number of comorbid conditions compared to patients who survived (Table 1). At baseline, no difference was found in the days since the onset of symptoms for patients who survived compared to those who died (Table 1).

**Table 1:** Demographic and clinical characteristic of the study population.

	**Study population (*****N*** **=** **65)**			**COVID-19 patients (*****N*** **=** **54)**		
	**COVID-19 patients, *N* = 54**	**Control patients *N* = 11**	***p*-value**	**Dead patients *N* = 16**	**Survivors** ***N* = 38**	***p*-value**
Gender *N* (%)			>0.9			>0.9
Male	40 (74%)	8 (73%)		12 (75%)	28 (74%)	
Female	14 (26%)	3 (27%)		4 (25%)	10 (26%)	
Age	65 (57, 73)	70 (66, 76)	0.2	72 (65, 78)	62 (55, 71)	0.004
Smoking habit *N* (%)						
Active smoker	0 (0)	3 (27%)	0.003	0 (0)	0 (0)	NA
Former smoker	16 (30%)	4 (36%)	0.725	7 (44%)	9 (24%)	0.2
BMI (kg/m^2^)	26.4 (24.2, 30.0)	24.8 (22.0, 27.1)	0.13	28.5 (26.4, 30.9)	26.0 (24.1, 29.4)	0.2
Number of Comorbidities/patients	1.00 (0.00, 3.00)	2.00 (1.50, 3.00)	0.12	3.00 (1.75, 4.00)	1.00 (0.00, 2.00)	0.004
Respiratory support at recruitment *N* (%)						
O_2_ only	11 (20%)	2 (18%)		2 (12%)	9 (24%)	
HFNC or NIV	16 (30%)	6 (54%)		4 (25%)	12 (31%)	
IV	27 (50%)	3 (27%)		10 (62%)	17 (45%)	
Days from symptoms onset to recruitment	9 (5–14)	5 (2–8)		10 (5–14)	8 (5–15)	0.60
Treatments *N* (%)						
Low molecular weight heparin	54 (100%)	11 (100%)	>0.9	16 (100%)	38 (100%)	>0.9
Antibiotics	47 (87%)	10 (90%)	>0.9	14 (88%)	33 (87%)	>0.9
Systemic corticosterods	37 (69%)	9 (81%)	>0.9	12 (75%)	25 (66%)	0.7
Antivirals	29 (54%)	NA	NA	7 (44%)	22 (58%)	0.5
Hydroxychloroquine	40 (74%)	NA	NA	11 (69%)	29 (76%)	0.7

*BMI, body mass index; HFNC, high flow nasal canula; NIV, non-invasive ventilation; IV, invasive ventilation*.

### Blood IFN-α and IFN-β Levels in COVID-19 Patients and Clinical Outcomes

**Figure 1:**
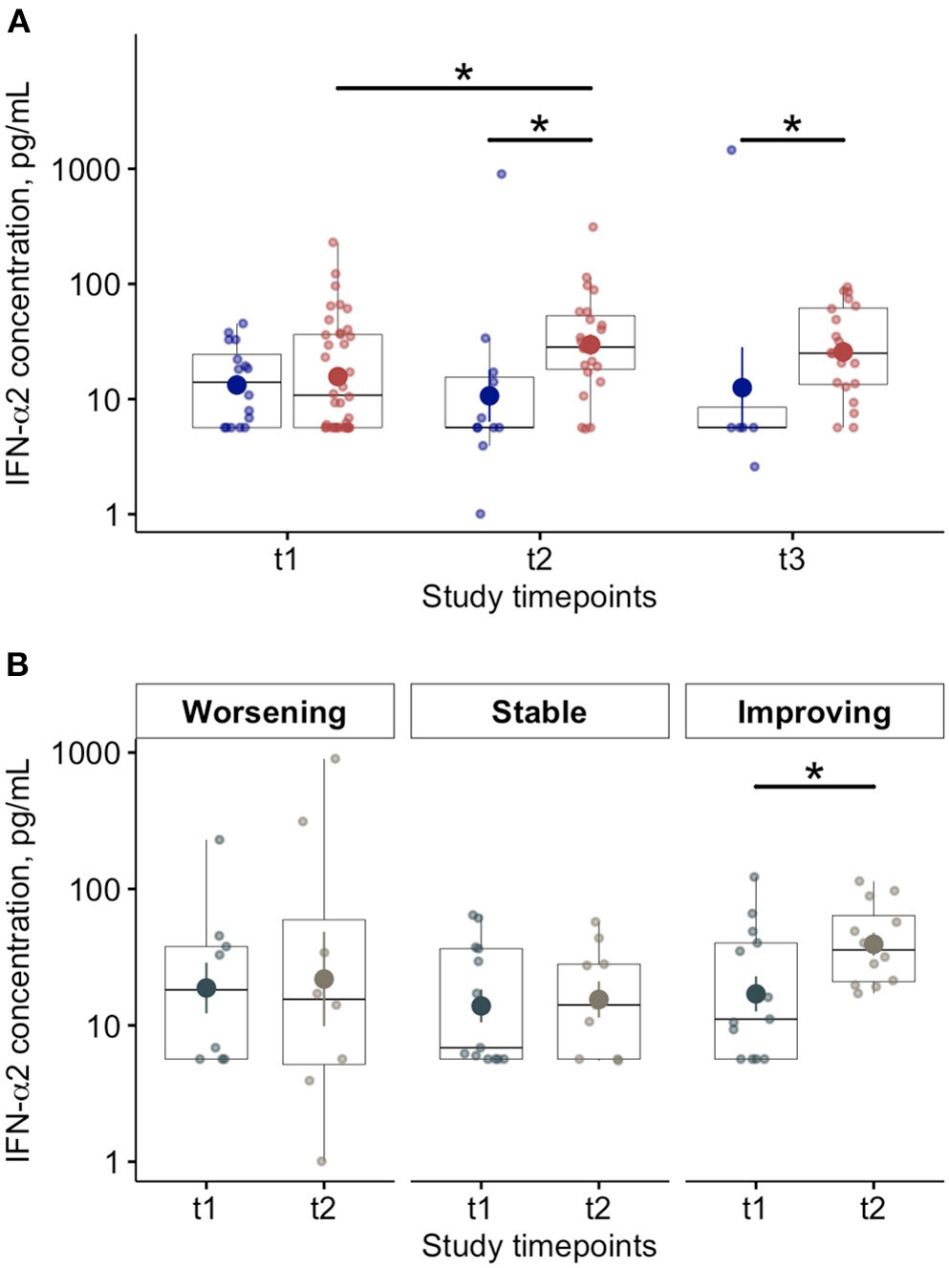
Blood Interferon (IFN)-α levels in COVID-19 patients and relationships with disease progression and clinical outcome. **(A)** Plasma IFN-α levels at baseline (t1) and at follow up (t2 and t3; 7 ± 2 day-interval between assessments) in patients who died (blue dots) or survived (red dots) during the study period. **(B)** Plasma interferon (IFN)-α levels at baseline (t1) and after 7 ± 2 days (t2) on the basis of worsening, stability or improving of the clinical manifestation of the disease (*p < 0.05).

**Figure 2:**
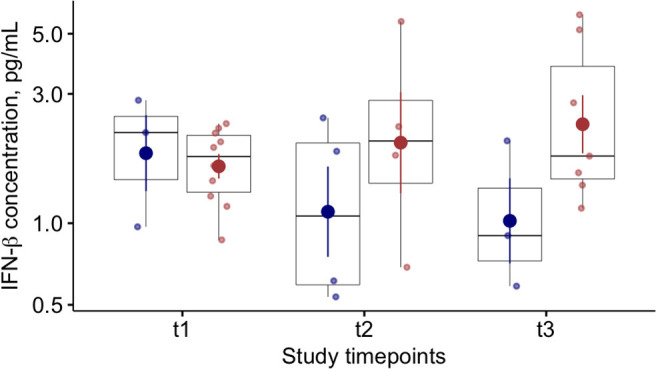
Blood Interferon (IFN)-β levels in COVID-19 patients and relationships with clinical outcome. Plasma IFN-β levels at baseline (t1) and at follow up (t2 and t3; 7 ± 2 day-interval between assessments) in patients who died (blue dots) or survived (red dots) during the study period.

At baseline (T1), we found lower blood levels of IFN-α in COVID-19 patients compared to controls [11 (6–34) vs. 42 (24–87) pg/ml *p* < 0.01)] (Table 2). Overall, COVID-19 data show lower levels of blood IFN-α in critically ill patients (requiring IV), compared to the COVID-19 patients requiring only oxygen supplementation (*p* = 0.03—Supplementary Figure 1A). Similarly, among critically ill COVID-19 patients requiring either IMV (*p* = 0.028) or NIMV (*p* = 0.061), higher levels of blood IFN-α were found in those who survived (Supplementary Figure 1B). Although baseline levels of IFN-α did not differ between survivors and non survivors COVID-19 patients, the values significantly increased over time in survivors, but not in COVID-19 patients with fatal evolution (Figure 1A). The reduction of severity of the manifestation of the COVID-19 between T1 and T2 (but not between T2 and T3) was paralleled by a significant increase of blood IFN-α levels (Figure 1B). Overall, blood IFN-β was undetectable in many samples (74% of totality of the samples). In the samples with detectable levels of IFN-β, the mean value was close to the detectability threshold (2.1 ± 0.3 pg/ml). At baseline, the percentage of blood samples with detectable IFN-β levels was 55% in the control group and it was as low as 26% in COVID19 patients (*p* = 0.06). Higher levels of plasma IFN-β levels were found at t2 and t3 in COVID-19 patients who survived compared to patients who died during the study period (Figure 2). At baseline (t1), no difference was found in the detectability of blood IFN-β in patients who worsen compared to patients who improved in the subsequent 7 ± 2 days. However, at t2 plasma IFN-β was undetectable in all the samples of patients who worsen compared to t1, while it was still detectable in 25% of samples of patients who clinically improved compared to baseline (Supplementary Figure 2).

**Table 2:** Blood cytokine levels and blood inflammatory cell counts at baseline (T1) in COVID-19 patients and controls.

	**COVID-19 patients, *N* = 54**	**Control patients, *N* = 11**	***p*-value**
IFN-α (pg/ml)	11 (6, 34)	42 (24, 87)	0.007
IFN-γ (pg/ml)	4 (2, 10)	5 (1, 132)	0.7
IL-1Ra (pg/ml)	17 (8, 53)	3 (2, 13)	0.005
IL-5 (pg/ml)	6 (3, 13)	1 (1, 1)	<0.001
IL-6 (pg/ml)	38 (10, 146)	8 (1, 33)	0.014
IL-10 (pg/ml)	9 (4, 36)	7 (2, 33)	0.6
IL-13 (pg/ml)	13 (5, 25)	5 (5, 9)	0.12
TNFα (pg/ml)	32 (22, 55)	11 (8, 34)	0.043
Total blood leucocytes (cells x10^3^/μl)	9.1 (6.8, 12.6)	12.0 (9.1, 14.7)	0.2
Blood lymphocites (cells x10^3^/μl)	0.83 (0.59, 1.04)	1.12 (0.52, 1.73)	0.3
Blood Neutrophils (cells x10^3^/μl)	7.9 (5.6, 10.2)	10.1 (5.8, 12.1)	0.2
Blood eosinophils (cells x10^3^/μl)	0.04 (0.00, 0.14)	0.00 (0.00, 0.06)	0.074

*[Data are expressed as Median (IQR)]*.

### Baseline Blood Inflammatory Cell Counts and Cytokine Levels

At baseline blood IL-1Ra, IL-5, IL-6, and TNF-alfa levels were significantly higher in COVID-19 compared to controls (Table 2). Overall, no differences were found in blood inflammatory cell counts between COVID-19 patients and controls (Table 2). Blood lymphocyte count was significantly lower, and blood IL-10 levels significantly higher in COVID-19 patients who died during the study period, compared to control patients [0.67 (0.47, 0.78) vs. 1.12 (0.52, 1.73) cells x10^3^/μl, *p* < 0.05; 33 (18, 53) vs. 7 (2, 9) pg/ml, *p* < 0.001, respectively].

### COVID 19 Survivors vs. Non-survivors: Blood Inflammatory Cells and Cytokine Levels Variations During Study Follow Up

**Figure 3:**
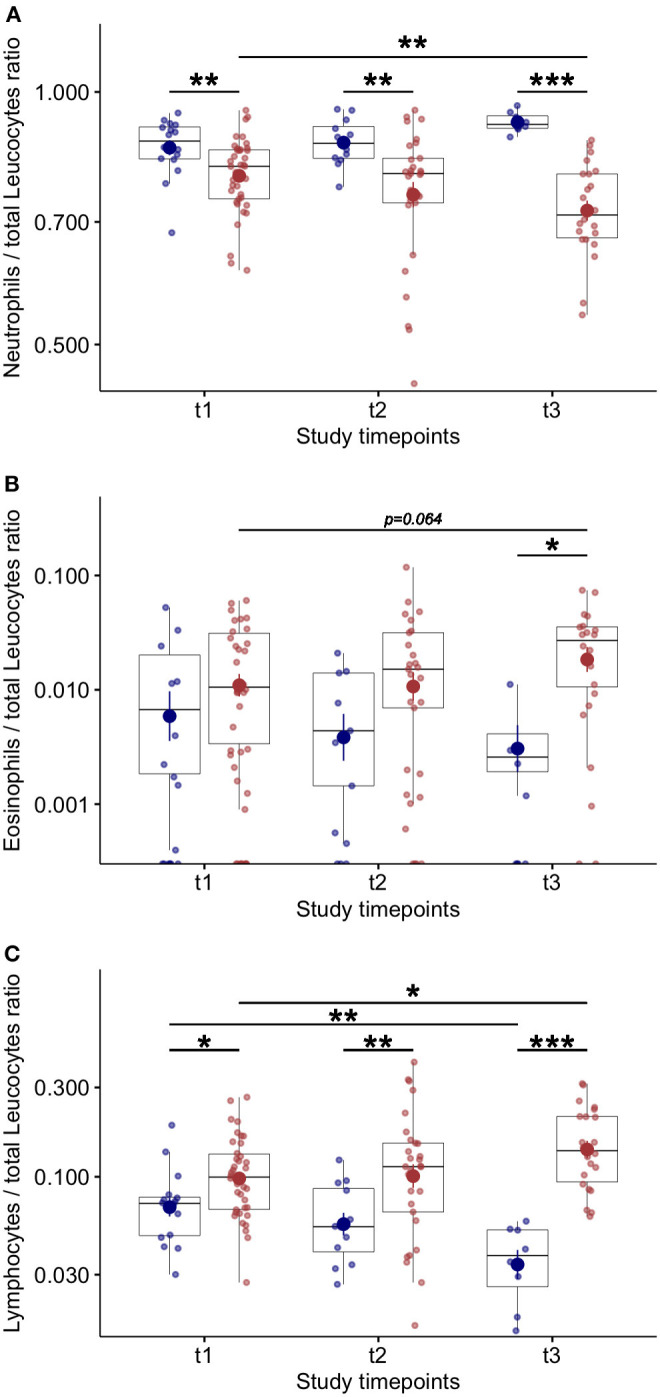
Blood inflammatory cell counts and variations during study follow up in COVID-19 patients. **(A)** Number of blood neutrophils over total leucocytes at baseline (t1) and at follow up (t2 and t3–7 ± 2 day-interval between assessments) in COVID-19 patients. **(B)** Number of blood eosinophils over total leucocytes at baseline (t1) and at follow up (t2 and t3–7 ± 2 day-interval between assessments) in COVID-19 patients. **(C)** Number of blood lymphocytes over total leucocytes at baseline (t1) and at follow up (t2 and t3–7 ± 2 day-interval between assessments) in COVID-19 patients. *p < 0.05; **p < 0.01; ***p < 0.001.

At baseline, no differences were found in blood neutrophil—(Figure 3A) and eosinophil—counts (Figure 3B) between COVID-19 patients who died compared to survivors. Lower blood lymphocytes counts were found at baseline in COVID-19 patients who died compared to survivors (Figure 3C). The eosinophil (Figure 3B) and lymphocyte counts (Figure 3C) increased during study follow up in COVID-19 patients who survived. Higher levels of blood eosinophils were found at T3 in patients who survived compared to patients who died (Figure 3B). In COVID-19 patients who died the number of blood lymphocyte significantly decreased during study follow up compared to survivors (Figure 3C) and the blood neutrophils counts were significantly higher at each study time point (Figure 3A). Also, significantly higher levels of C-reactive protein [17 (13–28) vs. 10 (3–16) mg/dl; *p* = 0.004] and procalcitonin were found in non survivors compared to survivors COVID-19 patients [0.74 (0.56–2.58) vs. 0.24 (0.17–0.38); *p* < 0.001)]. This data suggest that additional infective events related to impaired innate immunity can be associated with fatal outcomes (10).

**Figure 4:**
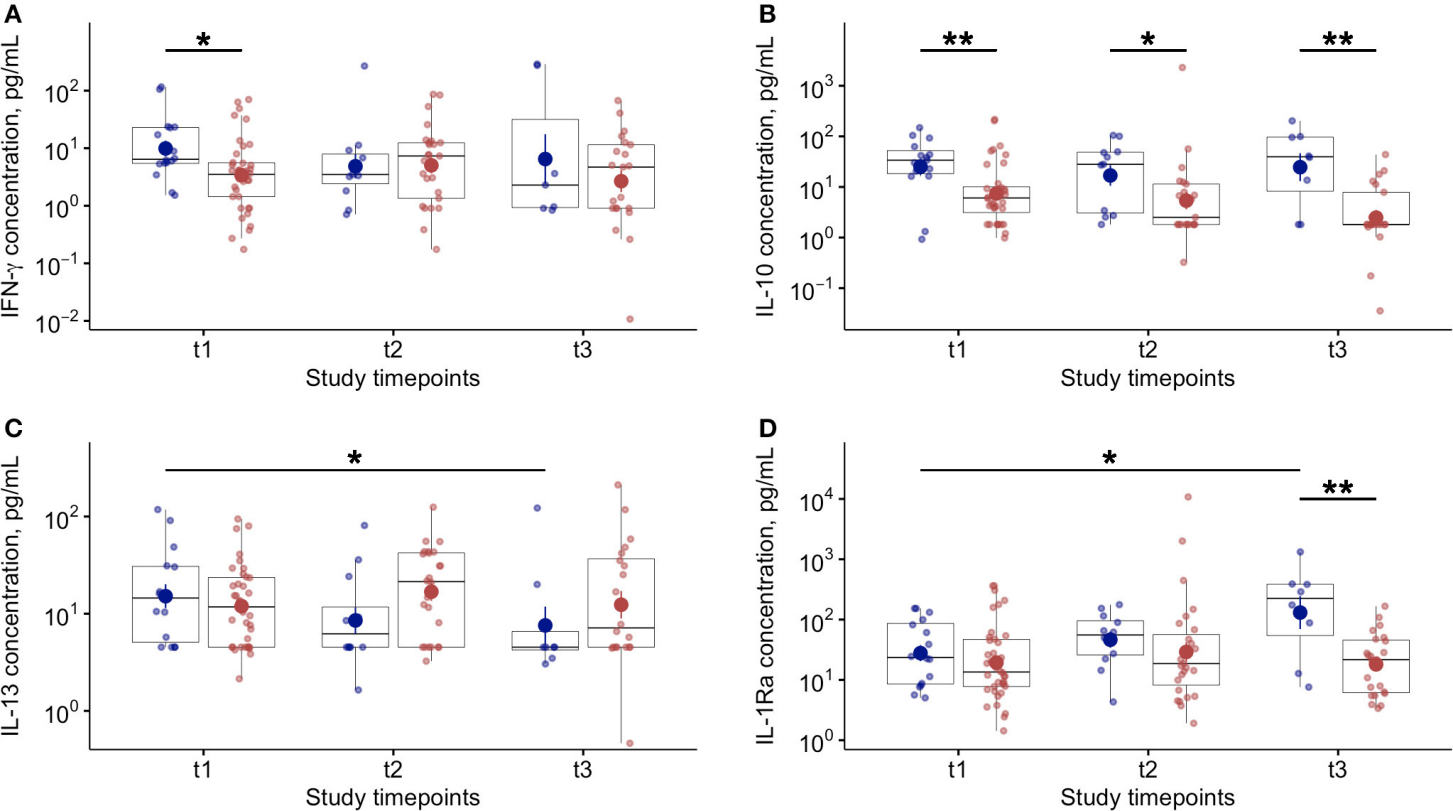
Blood cytokine levels and variations during study follow up in COVID-19 patients. **(A)** Plasma interferon (IFN)-γ levels at baseline (t1) and at follow up (t2 and t3–7 ± 2 day-interval between assessments) in patients who died (blue dots) or survived (red dots) during the study period. **(B)** Plasma interleukin (IL)-10 levels at baseline (t1) and at follow up (t2 and t3–7 ± 2 day-interval between assessments) in patients who died (blue dots) or survived (red dots) during the study period. **(C)** Plasma interleukin (IL)-13 levels at baseline (t1) and at follow up (t2 and t3–7 ± 2 day-interval between assessments) in COVID-19 patients. **(D)** Plasma interleukin (IL)-1Ra levels at baseline (t1) and at follow up (t2 and t3–7 ± 2 day-interval between assessments) in COVID-19 patients. In all panels, blue dots refer to COVID-19 patients who died, red dots refer to COVID-19 patients who survived during the study period. *p < 0.05; **p < 0.01.

Higher levels of blood IFN-γ were found at baseline in patients who died compared to survivors (Figure 4A). In line with previous studies (8, 11), we found a significant reduction of blood IL-6 levels in patients with favorable outcome during the study follow up (Supplementary Figure 3A). Notably, in COVID-19 patients the IL-10 levels were found significantly higher, at each time point, in patients who died compared to survivors (Figure 4B). In COVID-19 patients who died, blood IL-13 (Figure 4C) levels significantly decreased while IL-1Ra significantly increased at T3 compared to baseline (Figure 4D). No other variations in blood cytokine levels were found in COVID-19 patients during study follow up (Supplementary Figure 3).

### Correlations Between Blood IFN-α and Immunoinflammatory Biomarkers; A Network Analysis

**Figure 5:**
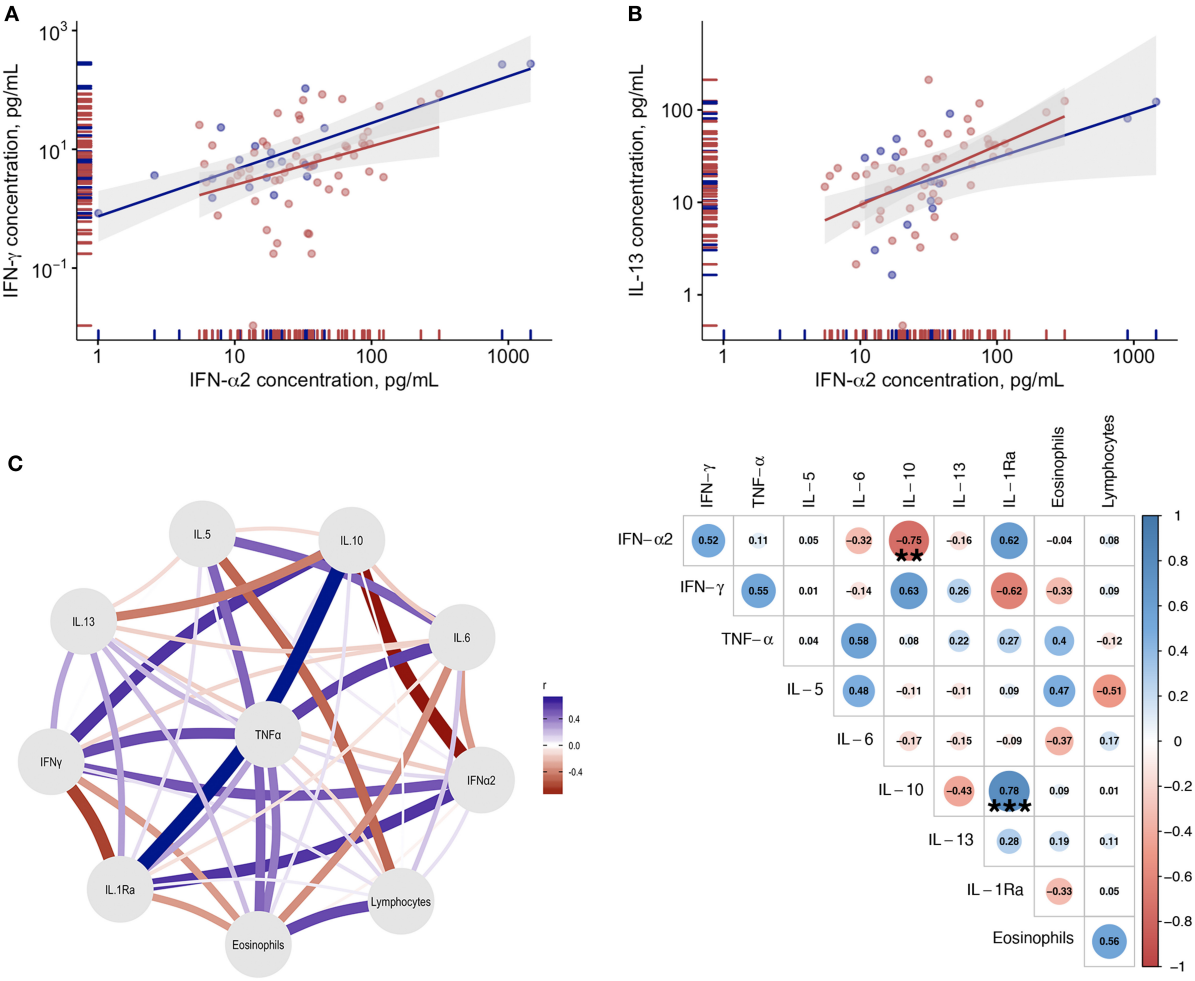
Correlations between blood IFN-α and immunoinflammatory biomarkers. **(A)** Correlation between plasma IL-13 levels and plasma IFN-α levels in COVID-19 patients. **(B)** Correlation between plasma IFN-γ levels and plasma IFN-α levels. Blue dots refer to COVID-19 patients who died, red dots refer to COVID-19 patients who survived during the study period. **(C)** Gaussian Graphical Model and corresponding correlation matrix of the tested biomarkers in COVID-19 patients with clinical improvement. In the correlation matrix the Spearman correlation coefficients are provided for variables correlations (blue for positive correlations and red for negative correlations). **Indicates statistical significance at the *p* < 0.01 level; ***p < 0.001.

When all time points were considered, no correlations were found between IFN-α levels and blood inflammatory cell counts (data not shown). Among the tested immunoinflammatory biomarkers, only IFN-γ levels (*r* = 0.47; *p* < 0.001—Figure 5A) and IL-13 (*r* = 0.53; *p* < 0.001—Figure 5B) significantly and directly correlated with blood IFN-α levels. The network analysis of the tested immunoinflammatory biomarkers revealed a significant indirect association between IL-10 and IFN-α levels (*r* = −0.749; *p* < 0.01), and a direct association between IL-10 and IL-1Ra levels (*r* = 0.779; *p* < 0.001—Figure 5C), in patients who clinically improved and survived.

## Discussion

Several studies highlighted the key role of inflammatory burst in the mechanisms of severe clinical manifestation of SARS-COV-2 infection (2, 12). However, pharmacologic interventions able to block specific inflammatory molecules led to limited favorable outcomes in COVID-19 patients. In our study we aimed to specifically and prospectively evaluate Type I IFN responses, i.e., a pivotal component of the early defense system against viral infection, in the clinical manifestation of COVID-19 patients presenting with respiratory failure. In particular, we evaluate blood IFN-α levels in relation to clinical outcomes and blood immune/inflammatory profile. Blood level of the type I IFN-β has been also evaluated to investigate in more depth the role of type I IFNs in COVID-19.

The data showed significantly lower levels of blood IFN-α, but higher levels of blood inflammatory cytokines, in COVID-19 patients compared to matched non-COVID patients. The increase in blood IFN-α levels in COVID-19 patients were directly associated with improvement in COVID-19 disease severity and greater survival. Notably, in patients who clinically improved and survived during the study, we found an inverse association between IL-10 and IFN-α levels.

In line with previous studies (7, 13) we found undetectable levels of IFN-β protein in a substantial proportion of COVID19 blood samples. However, a similar trend to IFN-α was found for IFN-β. Given the central role of IFN-β in antiviral immune responses, the undetectability of IFN-β in blood of COVID-19 patients can suggest for exogenous/pharmacological restoration of this molecule as a therapeutic option in these patients. Further studies are needed to better evaluate the role IFN-β in these patients.

Our results confirm previous cross-sectional studies showing lower levels of blood IFN-α in hospitalized COVID-19 patients compared to controls (14) and in critically ill COVID-19 patients compared to mild-moderate COVID-19 patients (7, 13). However, the longitudinal approach of our study together with the intense clinical characterization and the immune-inflammatory profiling allow to extend these observations by providing clinical information related longitudinally to role of type I IFN production to disease severity and interaction with inflammatory burst that characterized the disease.

The mechanisms of impaired type I IFN production in these conditions are largely unknown and can be related to host—but also microbe-specific mechanisms. The elderly comorbid patients are known to be the cohort of patients with higher risk of unfavorable outcomes related to SARS-COV-2 infection (15, 16). Aging has been associated with modifications in signaling mechanisms responsible for IFN production, leading to reduced IFN production (17). Recent studies reported the association between inborn errors in type I IFN-related genes (18–21)or the presence of auto-antibodies against type I IFN (in at least 10% of patients) and life-threatening COVID-19 conditions (19). On the other side, coronavirus (22) and specifically SARS-COV-2 infections (14, 23), have been shown to poorly induce IFN production; a mechanisms of possible viral immune escape. These immunological deficiencies can synergistically cooperate in a single patient leading to impaired IFN production, insufficient immune responses and more severe manifestation of COVID-19.

In addition, the virus-induced inflammatory response can lead to impaired/delayed innate immune responses (8, 24). Lymphopenia is a common clinical aspect of COVID-19 particularly in those who died (25, 26), as we report in our study. In the study we did not document a significant direct correlation between lymphocyte count and IFN-α levels. However, we found significant increased number of blood lymphocytes, through the study follow up, in patients who survived, which are also characterized by increase IFN-α production. Thus, these data suggest a parallelism between the lymphocytic response and the IFN production. Also, in COVID-19 patients who survived we found that the number of eosinophils (i.e., important cells of the antimicrobial immune responses) significantly increase and that, overall, Th1 cytokines (IFN-γ) paralleled type I IFN response. These findings further support the importance of an adequate immune response for favorable clinical outcomes in COVID-19.

At baseline, we found higher levels of blood Th2 inflammatory cytokines (IL-5 and IL-13) in the blood of COVID-19 patients compared to controls. The role of T2-cytokine in the context of COVID-19 is still largely unknown. A very recent study showed overexpression of Th2 cytokine in the lung of COVID-19 patients and proposed a role for Th2 cytokine in promoting lung damages in the context of COVID-19 (27). Not lastly, a negative interplay has been previously documented between Th2 inflammatory milieu and interferon responses (28). Thus, SARS-COV-2 induced T2 cytokines can contribute to lung damages and deranged immune responses in COVID-19. The complexity and misalignment of the immunological and inflammatory responses is highlighted by the concomitant reduction in blood IL-13 levels in COVID-19 patients who died during the study follow up and by the direct correlation between blood IL-13 and IFN-alfa levels, irrespective of the clinical outcome (death or survival). These data suggest a protective, rather than a negative, effect for Th2 cytokines (such as IL13) in the context of COVID-19.

Type I IFN and anti-inflammatory cytokines such as IL-10 and IL-1Ra have pleiotropic roles in the regulation of innate and adaptive immune responses. Type I IFNs can amplify innate immune responses by boosting natural killer and monocyte function and by preventing down-regulation of early immune responses by blocking IL-10 production in activated human monocytes (29). On the other hand, IL-10 can limit innate immune responses by inhibiting type I IFNs (30) and in cooperation with other anti-inflammatory cytokine, such as IL1Ra (31), can inhibit the development of adaptive immune response. Therefore, these anti-inflammatory cytokines can have deleterious effects in a context like COVID-19 patients. Indeed, in line with our findings, it has been shown that IL10 and IL1ra are significantly associated with COVID-19 severity and poor outcomes (32). Thus, the impaired Type I IFN production documented in critically ill patients does not hamper virus induced IL-10 production, which further impairs antiviral responses. However, in line with a previous study (33), we found that IL-1Ra and also IL-10, particularly in critically ill patients who died, are highly-expressed in SARS-CoV-infected subjects compared to control. Thus, because of the negative feedback of anti-inflammatory cytokines, overexpression of IL-1Ra IL-10 can lead to consequent impaired type I IFN responses.

In conclusion, this prospective observational study further supports the importance of deficient type I IFN responses to SARS-COV-2 infection in progression of COVID-19 toward a more severe and life-threatening manifestation. Interestingly, low levels of IFNs were found at baseline in COVID-19 patients irrespective from the clinical outcomes, suggesting that the SARS-COV-2 infection hampers the IFN-responses. However, these responses are not permanently abrogated or exhausted after the initial acute phase of the infection. The longitudinal analyses allowed to document a recover of the immune responses in COVID-19 patients in association to clinical improvement and favorable outcomes. Here we propose that the inflammatory milieu, particularly through the induction of anti-inflammatory cytokine such as IL-1Ra and Il-10, can lead to delayed/deranged innate immune responses. A recently published study explored, with a prospective longitudinal design similar to our study, the role of type I and III IFN responses in hospitalized COVID-19 patients (8). Consistently with our results the authors found profoundly impaired interferon production in COVID-19 patients compared to patients previously admitted because of severe influenza syndrome. At variance with our study the authors found that only a fraction of critically ill patients made IFN-α, while we found lower levels of blood IFN-α in patients requiring invasive ventilation compared to milder COVID-19 patients and a significant but delayed IFN-α production in COVID-19 patients who survived. The differences in the time sampling [early sampling by Galani et al. (8)] is crucial in the interpretation of our findings and differences in patients' characterization can also be partly explaining the different findings.

Our study is not without caveats. Firstly, it must be recognized that virtually all the available scientific evidence provided so far on the impaired interferon responses in COVID-19 patients, including our study, evaluated hospitalized patients. The immunological events that occur in the vast majority of COVID-19 patients not requiring hospitalization, are virtually unknown. Also, type I interferon are very rapidly and transiently induced antiviral molecules. Thus, earlier sampling from the onset of symptoms would be needed to fully characterize the role and the kinetic of interferon induction in SARS-COV-2 infection. Also, IFN signaling through IFN receptors results in transcriptional activation of a large set of genes called interferon-stimulated genes (ISGs). The protein products of ISGs control pathogen infections and shape the adaptive immune response (9). Thus, assessment of gene expression profiles of ISGs in blood cells of COVID19 patients are needed to further evaluate the role of IFN responses in COVID19. Finally, the sample size (particularly for the control group) is limited but not dissimilar to studies recently published exploring similar outcomes (7, 8). Furthermore, it is well known that during the first wave of the COVID outbreak a significant reduction in hospitalization and admission to ER for non-COVID acute conditions has occurred (34–36). These aspects led to a limited number of patients suitable for the control group.

The interferon deficiency in severe COVID-19 patients highlights the possibility of type I IFN modulation as a therapeutic approach in these patients. On this regard, preliminary observations showed that early (within 7-10 days from the onset of symptoms) subcutaneous injection of IFN- β1 on top of antiviral treatments, resulted in favorable outcomes including alleviation of symptoms and shortening in the duration if viral shedding in mild-to-moderate (37) but also reduced mortality in severe (38) COVID-19 patients. Indeed, nebulized IFN-β given to hospitalized COVID-19 has recently proved greater odds of improvement and more rapid recover from SARS-CoV-2 infection compared to placebo (39). Future studies are warranted to explore the effectiveness of this therapeutic approach in COVID-19.

## Data Availability Statement

The datasets presented in this article are not readily available because of local ethics constraints. Requests to access the datasets should be directed to the Corresponding author and to Comitato Etico di Area Vasta Emilia Centro (CE-AVEC) c/o Ufficio Ricerca e Innovazione Azienza Ospedaliero Universitaria di Ferrara Via Aldo Moro 8, 44124 Cona, Ferrara, Italy.

## Ethics Statement

The studies involving human participants were reviewed and approved by Comitato Etico Area Vasta Emilia Centro, Bologna, Italy. The patients/participants provided their written informed consent to participate in this study.

## Author Contributions

MC, GC, and SS: conceived and designed the research. LR, FT, LM, and OZ: acquired the data. FV, FF, and PR: performed laboratory analyses. LT: performed statistical analysis. MC and AP: drafted the manuscript. MC, AP, AF, RP, CV, NB, SJ, SS, and GC: made data interpretation and critical revision of the manuscript for key intellectual content. All authors contributed to the article and approved the submitted version.

## Conflict of Interest

MC reports grants, personal fees and non-financial support from Chiesi, personal fees and non-financial support from AstraZeneca, personal fees and non-financial support from Boehringer Ingelheim, personal fees and non-financial support from Alk-Abello, grants, personal fees and non-financial support from GlaxoSmithKline, personal fees and non-financial support from Novartis, personal fees and non-financial support from Zambon, grants from University of Ferrara - Italy, outside the submitted work. AP reports grants, personal fees, non-financial support and other from GlaxoSmithKline, grants, personal fees and non-financial support from AstraZeneca, grants, personal fees, non-financial support and other from Boehringer Ingelheim, grants, personal fees, non-financial support and other from Chiesi Farmaceutici, grants, personal fees, non-financial support and other from TEVA, personal fees, non-financial support and other from Mundipharma, personal fees, non-financial support and other from Zambon, personal fees, non-financial support and other from Novartis, grants, personal fees and non-financial support from Menarini, personal fees, non-financial support and other from Sanofi/Regeneron, personal fees from Roche, grants from Fondazione Maugeri, grants from Fondazione Chiesi, personal fees from Edmondpharma, outside the submitted work. SJ reports personal fees from Virtus Respiratory Research, personal fees from Myelo Therapeutics GmbH, personal fees from Concert Pharmaceuticals, personal fees from Bayer, personal fees from Synairgen, personal fees from Novartis, personal fees from Boehringer Ingelheim, personal fees from Chiesi, personal fees from Gerson Lehrman Group, personal fees from resTORbio, personal fees from Bioforce, personal fees from Materia Medical Holdings, personal fees from PrepBio Pharma, personal fees from Pulmotect, personal fees from Virion Health, personal fees from Lallemand Pharma, personal fees from AstraZeneca, outside the submitted work and has a patent Wark PA, Johnston SL, Holgate ST, Davies DE. Anti-virus therapy for respiratory diseases. UK patent application No. GB 0405634.7, 12 March 2004. with royalties paid, a patent Wark PA, Johnston SL, Holgate ST, Davies DE. Interferon-Beta for Anti-Virus Therapy for Respiratory Diseases. International Patent Application No. PCT/ GB05/50031, 12 March 2004. with royalties paid, and a patent Davies DE, Wark PA, Holgate ST, Johnston SL. Interferon Lambda therapy for the treatment of respiratory disease. UK patent application No. 6779645.9, granted 15th August 2012. licensed. GC reports grants and personal fees from Astrazeneca, grants and personal fees from Boston Scientific, grants and personal fees from SMT, grants and personal fees from Eukon, grants and personal fees from Daiichi Sankyo, grants and personal fees from Menarini, outside the submitted work. The remaining authors declare that the research was conducted in the absence of any commercial or financial relationships that could be construed as a potential conflict of interest.
