# Supplementary figures and images for: Blood Interferon-α Levels and Severity, Outcomes, and Inflammatory Profiles in Hospitalized COVID-19 Patients
Source: Front Immunol. 2021 Mar 9;12:648004. doi: 10.3389/fimmu.2021.648004 (PMC7985458; doi:10.3389/fimmu.2021.648004)

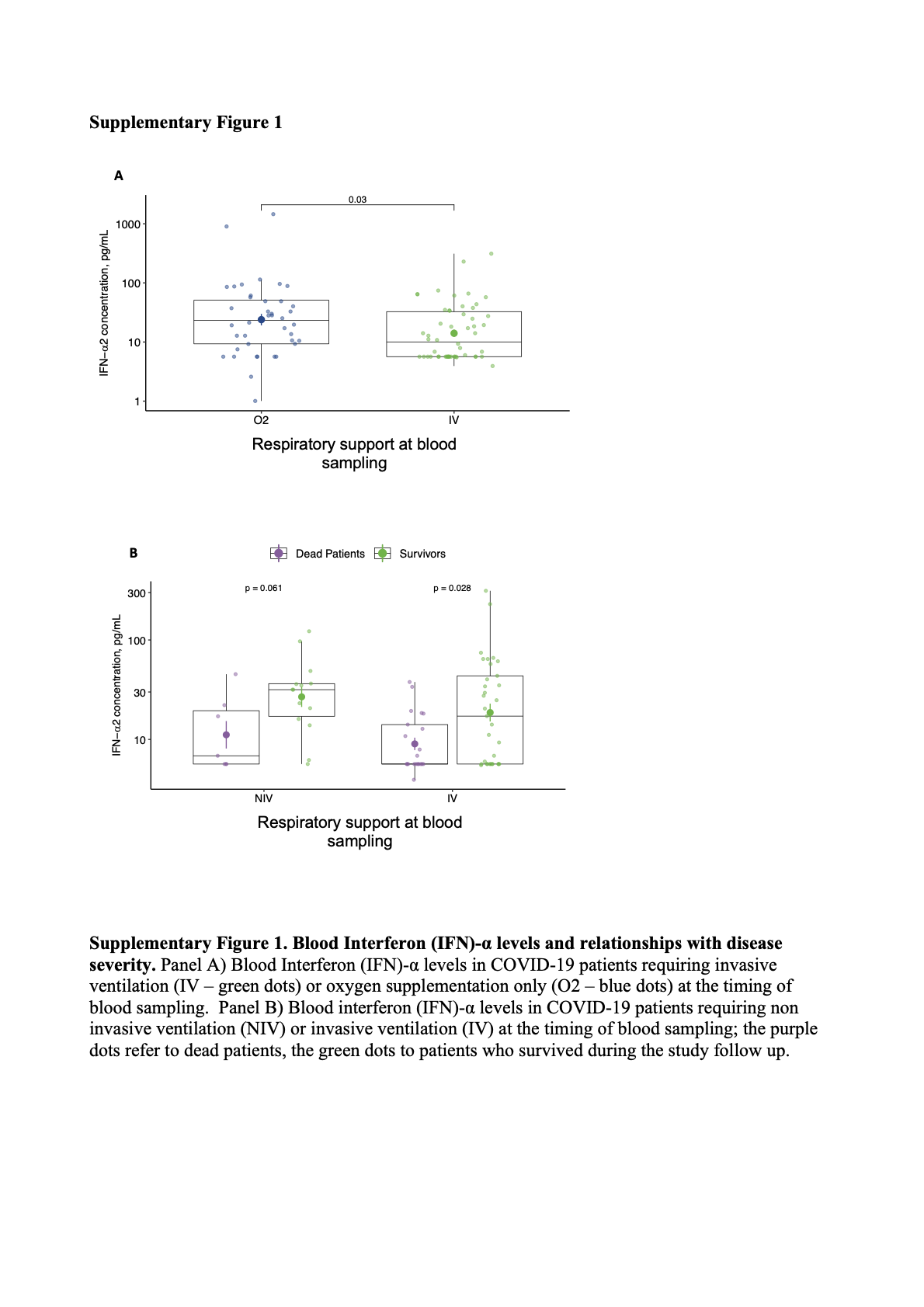

Supplement: Supplementary file 1 [file Image_1.TIFF]

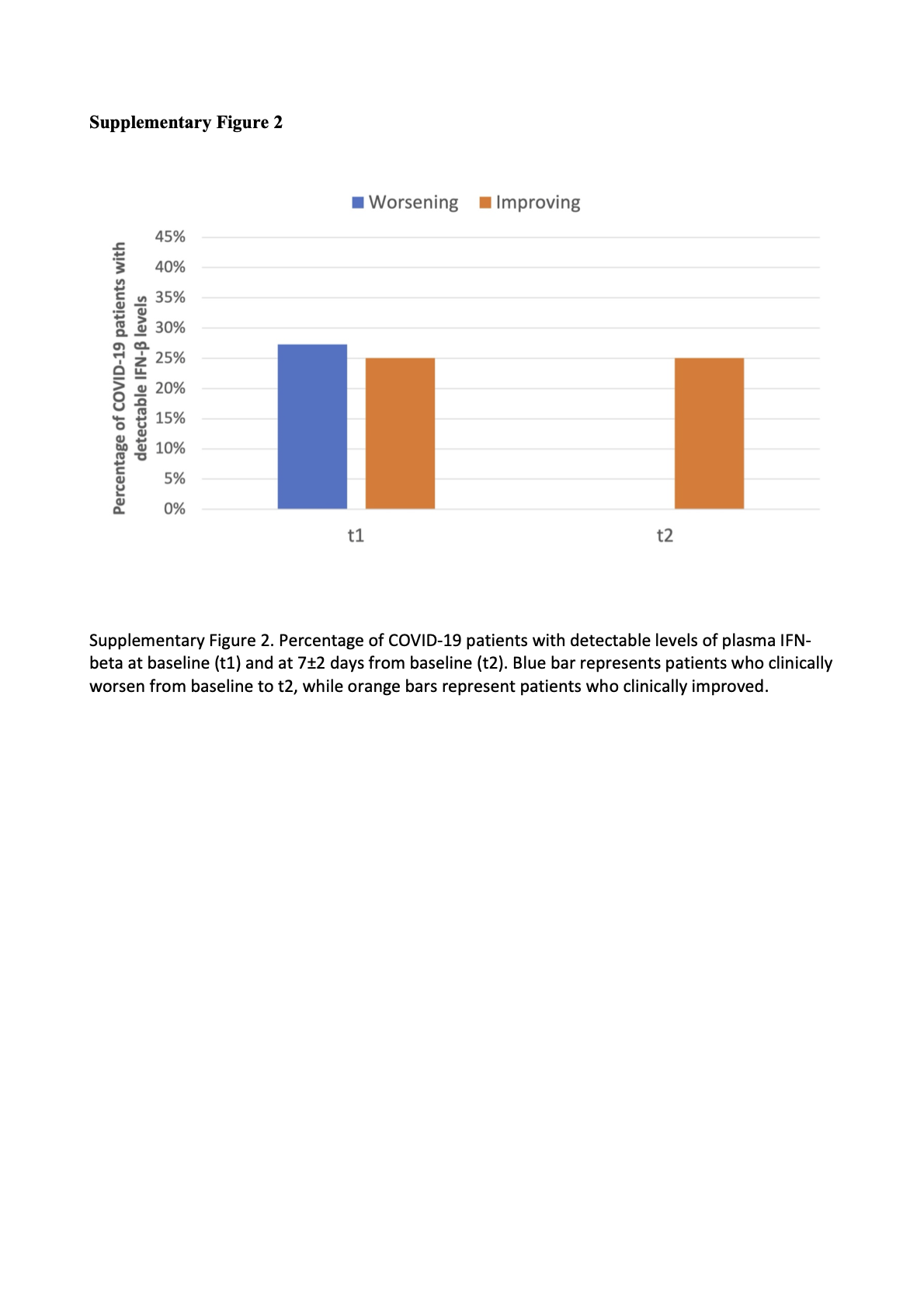

Supplement: Supplementary file 2 [file Image_2.TIFF]

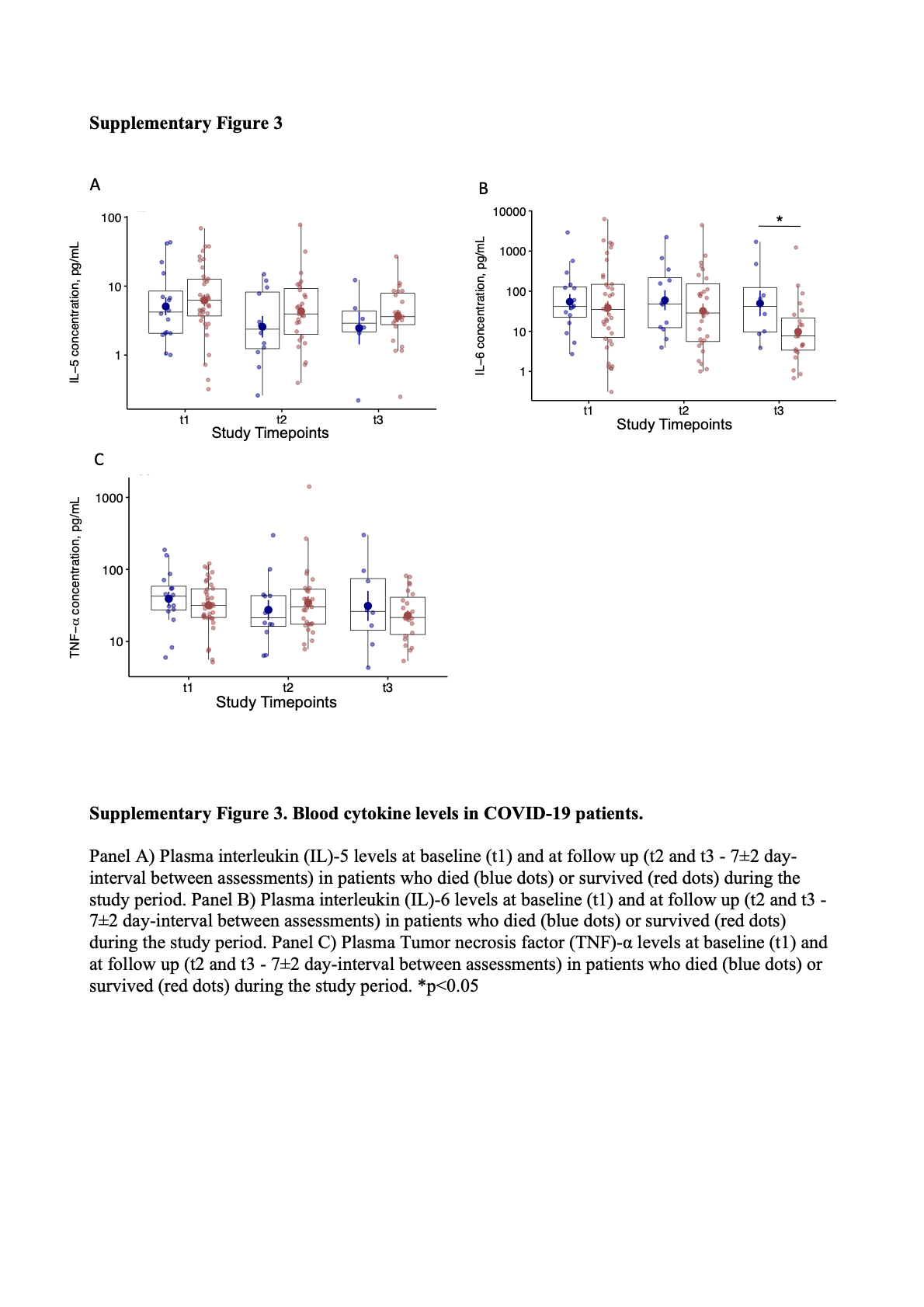

Supplement: Supplementary file 3 [file Image_3.TIFF]
